# Barriers to Guideline-Based Management of Esophageal Carcinoma: A Narrative Review With Special Reference to North East India

**DOI:** 10.7759/cureus.111199

**Published:** 2026-06-20

**Authors:** Sumit Kumar, Sandeep Ghosh, Deiwakor Chyrmang, Biswajit Dey, Tamajyoti Ghosh, Binoy Singh

**Affiliations:** 1 Radiation Oncology, North Eastern Indira Gandhi Regional Institute of Health and Medical Sciences, Shillong, IND; 2 Surgical Oncology, North Eastern Indira Gandhi Regional Institute of Health and Medical Sciences, Shillong, IND; 3 Pathology, North Eastern Indira Gandhi Regional Institute of Health and Medical Sciences, Shillong, IND; 4 Neurosurgery, North Eastern Indira Gandhi Regional Institute of Health and Medical Sciences, Shillong, IND; 5 Neurosurgery, All India Institute of Medical Sciences, Raipur, Raipur, IND

**Keywords:** cancer care delivery, guideline-concordant care, implementation barriers, north east india, sophageal carcinoma

## Abstract

Esophageal carcinoma remains one of the most lethal malignancies worldwide. It poses a significant public health challenge in India, particularly in the North-Eastern region, where the disease burden is among the highest in the country. Contemporary management has evolved into a multidisciplinary, stage-directed, and increasingly biomarker-informed approach. This approach incorporates accurate staging, nutritional assessment, neoadjuvant or perioperative therapy, surgery, definitive chemoradiotherapy, immunotherapy, palliation, and structured follow-up. However, the successful implementation of guideline-based care requires coordinated delivery of multiple healthcare services across the cancer care continuum.

This narrative review examines barriers to guideline-based management of esophageal carcinoma, with special reference to North East India. A literature search of PubMed, Google Scholar, major oncology guidelines, and relevant regional publications was conducted to identify evidence on diagnosis, staging, treatment delivery, nutritional support, multidisciplinary care, access to surgery and radiotherapy, financial challenges, and healthcare-system barriers. Findings were synthesized along the patient care pathway from symptom onset to long-term follow-up.

Available evidence suggests that substantial implementation gaps exist despite advances in evidence-based management. Delayed presentation, malnutrition, geographical isolation, poor transportation infrastructure, linguistic diversity, financial toxicity, fragmented referral pathways, limited access to specialized oncology services, treatment authorization requirements, and loss to follow-up contribute to deviations from guideline-concordant care. These challenges are particularly relevant in North East India, where difficult terrain, dispersed populations, and high disease incidence place additional strain on healthcare delivery systems.

The review proposes practical, locally adapted strategies to improve implementation, including dysphagia-to-treatment fast-track pathways, nutrition-first assessment, multidisciplinary coordination, patient navigation services, language-sensitive counselling, accommodation and food support, tele-follow-up, and alignment of public healthcare packages with contemporary evidence-based treatment requirements. Improving outcomes in esophageal carcinoma will require not only advances in clinical management but also systematic efforts to ensure that guideline-recommended care is accessible, affordable, and deliverable within regional healthcare settings.

## Introduction and background

Esophageal carcinoma is a malignancy in which the evidence-implementation gap is highly visible. Although contemporary guidelines define clear pathways for diagnosis, staging, multimodality treatment, and follow-up, many patients are unable to receive or complete guideline-concordant care in routine practice. Globally, esophageal cancer accounted for an estimated 511,054 new cases and 445,391 deaths in 2022, reflecting its aggressive clinical course and high mortality-to-incidence ratio [1].

The burden is especially relevant in North East India, where upper gastrointestinal and upper aerodigestive tract malignancies are common. Population-based cancer registry data show that esophageal cancer is the leading cancer among males in pooled North-Eastern region (NER) data, contributing 13.6% of male cancers [2]. East Khasi Hills district of Meghalaya has reported one of the highest age-adjusted incidence rates of esophageal cancer in India, with rates of 75.4 per 100,000 men and 33.6 per 100,000 women [3].

Guideline-based management refers to care that is aligned with established evidence-based recommendations for diagnosis, staging, treatment, and follow-up. Guideline-based management is complex because it depends on a time-sensitive sequence of care rather than a single therapeutic decision. Indian consensus guidance provides a national framework, while contemporary National Comprehensive Cancer Network (NCCN), European Society of Medical Oncology (ESMO), and American Society of Clinical Oncology (ASCO) recommendations emphasize accurate staging, multidisciplinary evaluation, nutritional assessment, and selection of neoadjuvant chemoradiotherapy, perioperative chemotherapy, definitive chemoradiotherapy, surgery, systemic therapy, adjuvant therapy, or palliation [4-8]. The evidence base has expanded through trials such as Chemotherapy for Esophageal Cancer Followed by Surgery Study (CROSS), fluorouracil, leucovorin, oxaliplatin, and docetaxel (FLOT) 4, and CheckMate 577, which established roles for neoadjuvant chemoradiotherapy, perioperative FLOT chemotherapy, and adjuvant nivolumab in selected patients [9-11].

However, expansion of evidence-based options has widened the gap between what is recommended and what is deliverable. Paclitaxel-carboplatin-based chemoradiotherapy may be accessible through government-supported pathways [12]. In contrast, newer regimens, immunotherapy, biomarker testing, advanced imaging, nutritional interventions, intensive supportive care, and perioperative surgical requirements may not be uniformly available or covered by insurance [12]. Thus, implementation depends not only on clinician knowledge but also on infrastructure, package coverage, approval pathways, affordability, referral coordination, and patient fitness.

North East India provides a distinct setting for examining this gap. Hilly terrain, poor road connectivity, dispersed rural populations, prolonged travel time to tertiary oncology centers, socioeconomic heterogeneity, and linguistic diversity may adversely affect timely diagnosis, treatment acceptance, daily radiotherapy attendance, surgical referrals, toxicity reporting, and long-term follow-up [2, 13, 14]. The novelty of this narrative review lies in its focus on the evidence-implementation gap in esophageal cancer care in North East India. Rather than concentrating solely on disease burden or treatment advances, it examines barriers across the care pathway and highlights region-specific implementation strategies to improve access to guideline-concordant care. This review, therefore, examines barriers to guideline-based management of esophageal carcinoma with special reference to North East India and proposes locally adapted strategies to make evidence-based care practically deliverable.

## Review

Materials and methods

This article is a narrative review with a regional perspective. A literature search was performed using PubMed, Google Scholar, and major oncology guideline sources for articles published between January 2010 and May 2026. Search terms included combinations of 'esophageal cancer', 'oesophageal cancer', 'guideline-based management', 'multimodality treatment', 'patient navigation', 'chemoradiotherapy', 'perioperative chemotherapy', 'nutrition', 'dysphagia', 'multidisciplinary team', 'barriers', healthcare access', 'radiotherapy', 'esophagectomy', 'palliative care', 'low- and middle-income countries', 'India', and 'North East India'.

International guidelines, landmark trials, review articles, observational studies, implementation-focused publications, government sources, and North East India-specific cancer-care literature were considered. Articles were selected if they addressed diagnosis, staging, treatment sequencing, nutritional issues, multidisciplinary care, radiotherapy or chemotherapy delivery, surgical access, financial or geographic barriers, palliative care, follow-up, or cancer-care delivery in resource-limited settings. Conference abstracts and literature not relevant to esophageal cancer care or oncology service implementation were excluded.

Findings were synthesized narratively along the esophageal cancer care pathway, from symptom onset and diagnosis to staging, multidisciplinary decision-making, treatment delivery, surgery or definitive chemoradiation, palliation, and follow-up. Given the limited region-specific literature available, relevant publications from the authors’ institution and collaborative work conducted in North East India were also included where appropriate. These studies provide insights into esophageal cancer care in one of India's highest-incidence regions and reflect local clinical experience. No institutional patient-level data were collected or analyzed, and formal Preferred Reporting Items for Systematic Reviews and Meta-Analyses (PRISMA) methodology, quantitative pooling, and risk-of-bias assessment were not performed.

Guideline-based management of esophageal carcinoma: what is expected?

Guideline-based management of esophageal carcinoma has evolved into a multidisciplinary, stage-directed, and biomarker-informed pathway [4-8]. Indian consensus guidance provides an India-focused framework, while contemporary NCCN, ESMO, and ASCO recommendations incorporate recent advances such as neoadjuvant chemoradiotherapy, perioperative FLOT-based chemotherapy, definitive chemoradiotherapy, adjuvant nivolumab after residual disease following neoadjuvant chemoradiotherapy and surgery, and immunotherapy- or biomarker-directed treatment in recurrent or metastatic disease [4-8, 10].

Therefore, modern guideline-based care requires timely diagnosis, accurate staging, nutritional assessment, multidisciplinary team (MDT) discussion, treatment sequencing, biomarker testing where indicated, access to radiotherapy, surgery, systemic therapy, palliation, and structured follow-up. MDT care involves collaborative decision-making among specialists from different disciplines to optimize patient management. The major components of contemporary guideline-based management across different clinical settings are summarized in Table 1.

**Table 1:** Contemporary guideline-based management of esophageal carcinoma across clinical settings MDT: Multidisciplinary team; SCC: Squamous cell carcinoma; GEJ: Gastroesophageal junction; NACTRT: Neoadjuvant chemoradiotherapy; GI: Gastrointestinal; CECT: Contrast enhanced computed tomography; PET-CT: Positron emission tomography-computed tomography; EUS: Endoscopic ultrasound; ECOG: Eastern Cooperative Oncology Group; CRT: Chemoradiation therapy; FLOT: Fluorouracil, leucovorin, oxaliplatin and docetaxel; PD-L1: Programmed Death-Ligand 1; HER2: Human epidermal growth factor receptor 2; MSI: Microsatellite instability; MMR: Mismatch repair; RT: Radiotherapy; ICMR: Indian Council of Medical Research; NCCN: National Comprehensive Cancer Network; ESMO: European Society of Medical Oncology; ASCO: American Society of Clinical Oncology; CROSS: Chemoradiotherapy for oesophageal cancer followed by surgery study; NCI: National Cancer Institute; PDQ: Physical data query Source references: [4-11].

Clinical setting	Expected guideline-based management	Key evidence/guideline basis
Diagnosis	Upper GI endoscopy with biopsy; histological confirmation as SCC or adenocarcinoma	ICMR, NCCN, ESMO
Staging	CECT chest/abdomen +/- pelvis; PET-CT/EUS where feasible; bronchoscopy for selected upper/mid-thoracic tumors	NCCN, ESMO
Baseline assessment	ECOG status, comorbidities, pulmonary/cardiac fitness, nutrition, and dysphagia assessment	NCCN, ESMO, ASCO
MDT decision	Joint planning by surgery, radiation oncology, medical oncology, radiology, pathology, nutrition, and palliative care	NCCN, ESMO
Early mucosal disease	Endoscopic resection in carefully selected early lesions; surgery or additional therapy based on risk features	NCCN
Locally advanced resectable SCC	Neoadjuvant chemoradiotherapy followed by surgery, or definitive CRT in selected patients	CROSS, NCCN, ESMO, ASCO
Locally advanced adenocarcinoma/GEJ	Preoperative CRT or perioperative chemotherapy; FLOT-based therapy in suitable patients	FLOT4, NCCN, ESMO, ASCO
Inoperable/unresectable non-metastatic disease	Definitive concurrent chemoradiotherapy where feasible	NCCN, ESMO, ASCO
Residual disease after NACTRT + surgery	Consider adjuvant nivolumab in eligible patients	CheckMate 577, NCCN, ESMO
Recurrent/metastatic disease	Systemic therapy based on histology, performance status, PD-L1, HER2, MSI/MMR and other biomarkers where applicable	NCCN, ESMO
Palliation/supportive care	Dysphagia palliation, feeding support, palliative RT, pain control, nutrition, and early palliative care	NCCN, ESMO, NCI PDQ
Follow-up	Surveillance, toxicity assessment, nutritional rehabilitation, and symptom-directed care	NCCN, ESMO

This expected pathway provides the benchmark against which real-world barriers to guideline implementation can be evaluated.

The evidence-implementation gap

The benefit demonstrated in modern esophageal cancer trials assumes timely staging, nutritional fitness, access to chemotherapy, radiotherapy, surgery, pathology, biomarker testing, postoperative decision-making, and structured follow-up [4, 5, 8, 9]. In routine practice, these assumptions are often challenged. A 2024 study evaluating foregut cancer care pathways reported that over one-third of patients (34.1%) did not receive guideline-concordant treatment, largely attributable to delays across initial symptom evaluation, diagnostic workup, oncology referral, and treatment initiation [15].

In esophageal cancer, this gap is amplified by dysphagia, weight loss, dehydration, sarcopenia, and malnutrition, which may reduce tolerance to chemotherapy, chemoradiotherapy, and surgery even before treatment begins. In addition, the increasing incorporation of immunotherapy and multimodality treatment has also introduced new toxicity profiles and management challenges [16]. This assumes additional importance in high-incidence regions such as Meghalaya, where regional studies have demonstrated aggressive clinicopathological and molecular characteristics in esophageal cancer [17]. Structured nutrition management during concurrent chemoradiotherapy has been reported to improve nutritional status and reduce treatment-related toxicity [18].

India's publicly funded health insurance framework is decentralized, with several states augmenting the central 'Ayushman Bharat Pradhan Mantri Jan Arogya Yojana' (AB-PMJAY) scheme. Assam was the first state in Eastern India to join the AB-PMJAY scheme [19]. In Meghalaya, around 55% of the eligible population is enrolled in the Megha Health Insurance Scheme (MHIS), which is implemented in direct convergence with AB-PMJAY [20]. These publicly funded schemes, like AB-PMJAY, have enhanced access to cancer care by covering chemotherapy, radiotherapy, and surgical oncology [12]. Despite incorporation into contemporary treatment guidelines, access to immunotherapy and targeted therapies may remain limited across the regions. Moreover, cancer treatment requires approval for a multimodal treatment plan [12]. Financial constraints remain significant, and both direct and indirect costs, such as diagnostics, medicines, travel, lodging, and food, continue to impact treatment initiation and adherence [21]. Therefore, the implementation challenge extends beyond guideline recommendations to whether the required components of care are available, affordable, approved, and practically deliverable within the local healthcare system.

Barriers across the care pathway: a North East India perspective

Barriers in North East India are best conceptualized as pathway attrition, wherein patients may experience delays, treatment modifications, or loss to follow-up at successive stages ranging from diagnosis and staging to multidisciplinary decision-making, treatment initiation, reassessment, surgery, adjuvant therapy, palliation, and long-term follow-up [22]. Diagnostic delays may also occur because esophageal carcinoma can occasionally present with atypical manifestations, further complicating timely recognition and referral [23]. The region is not uniform; it includes eight states with different geography, languages, referral routes, and oncology access. Thus, despite substantial inter-state variation, the broader patterns of pathway attrition and barriers to guideline-concordant care are relevant across the North-Eastern region [2, 3].

A combined pathway and stakeholder perspective is valuable because the same underlying barrier may manifest differently across healthcare actors. For patients, it may involve challenges related to travel, language, accommodation, nutrition, fear, or financial toxicity; for clinicians, it may translate into malnutrition, incomplete staging, poor treatment tolerance, missed surgical windows, or interrupted therapy; whereas for hospitals and payers, it may present as package authorization delays, capacity constraints, and coordination failures across the care continuum [2, 3, 22]. To illustrate where implementation gaps occur, key barriers identified from published literature are mapped along the esophageal cancer care pathway in Table 2.

**Table 2:** Barriers to guideline-based esophageal cancer care across the patient pathway in North East India CRT: Chemoradiotherapy; AAR: Age-adjusted rate; NER: North-Eastern region; CCRT: Concurrent chemoradiotherapy; PM-JAY: Pradhan Mantri Jan Arogya Yojana; NACTRT: Neoadjuvant chemoradiotherapy; NACT: Neoadjuvant chemotherapy Source references: [2, 3, 12, 15, 18, 21, 24-26].

Pathway point	Published evidence/data	Stakeholder most affected	Implementation impact
Regional disease burden	Esophageal cancer contributes 13.6% of male cancers and 7.0% of female cancers in pooled North-Eastern region data.	Regional planners / tertiary centres	Need for a dedicated regional esophageal cancer pathway rather than episodic referral.
High-incidence pockets	East Khasi Hills reported AAR 75.4/100,000 men and 33.6/100,000 women.	Patients/referral hospitals	High-risk districts may overload tertiary pathways if early triage is weak.
Pre-diagnosis delay	Meghalaya qualitative data link delays to stigma, misconceptions, self-management, preference for traditional medicine, financial barriers, and health-system factors.	Patients/families	Late presentation with dysphagia, weight loss, and reduced treatment fitness.
Access to cancer institutes	A two-state NER study identified transportation, accommodation, infrastructure shortages, limited awareness, financial stress, family decision-making, and alternative medicine use.	Patients/caregivers	Repeated visits for staging, CRT, surgery, and follow-up become difficult.
Guideline-concordant care	In foregut cancers, 34.1% did not receive guideline-concordant treatment; delays occurred across workup, oncology visits, and treatment initiation.	Clinicians/systems	Guideline failures often happen due to delays in transition rather than just a lack of knowledge.
Post-neoadjuvant transition	Foregut attrition after neoadjuvant therapy may result from deconditioning, loss to follow-up, healthcare delivery gaps, and refusal of surgery.	Surgeons / MDT	Patients may complete NACTRT/NACT but fail to reach esophagectomy or adjuvant decision.
Nutrition and CRT tolerance	Whole-course nutrition management during esophageal CCRT improves nutritional status and reduces treatment-related toxicity.	Radiation/medical oncology teams	Nutrition is a treatment-readiness requirement, not only supportive care.
Communication-sensitive care	NER spans multiple languages, dialects, and tribal communities; Meghalaya data show stigma and misconceptions affect care seeking.	Patients/providers	Poor communication affects consent, toxicity reporting, nutrition advice, and follow-up.
Financial and indirect costs	Indian cancer-care journey literature identifies travel, lodging, food, and treatment-related disruption as major burdens.	Patients/caregivers	Cost can interrupt care even when treatment itself is subsidized.
Package and policy gap	PM-JAY covers chemotherapy, radiotherapy, and surgical oncology, but cancer packages require pre-authorization.	Government/payer/hospital	Modern components such as biomarker testing, immunotherapy, advanced staging, and feeding procedures may outpace package coverage.

Beyond the structural and logistical barriers summarized above, sociocultural factors may also influence healthcare-seeking behaviour and timely engagement with cancer services in North East India. Traditional beliefs and preference for indigenous healing practices may contribute to delayed healthcare seeking in some communities [24, 25]. Studies from Meghalaya have reported that patients may initially seek advice from traditional healers or use local remedies before accessing formal cancer services [14, 24]. Cultural notions in some communities that illness may result from a figurative “poison” or ill intent may further influence illness perceptions and healthcare-seeking behaviour [24]. Similar observations regarding limited awareness, family decision-making, and alternative medicine use have also been reported from other North-Eastern states [25].

Thus, barriers should not be framed simply as poor compliance or lack of facilities. They represent a multi-stakeholder implementation gap in which patient vulnerability, provider limitations, hospital coordination, payer coverage, and regional geography interact across the care pathway.

Strategies for improving implementation

Studies from North East India show that local interventions are essential because poor infrastructure, financial constraints, and geographical barriers make it difficult for people to access cancer care. cultural beliefs and a lack of awareness further delay patients from getting the medical treatment they need [24, 25]. In North East India, implementation strategies should focus on practical patient support, such as language-sensitive counselling, affordable food and lodging, and follow-up tracking. Additionally, these strategies must streamline care through fast-track diagnostics, nutrition-first assessments, MDT coordination, and package support. Potential implementation strategies tailored to the North East Indian context are summarized in Table 3.

**Table 3:** Locally adapted strategies to improve implementation of guideline-based esophageal cancer care PM-JAY: Pradhan Mantri Jan Arogya Yojana; OPD: Outpatient department; RT: Radiotherapy; CT: Computed tomography; PET-CT: Positron emission tomography-computed tomography; MDT: Multidisciplinary team; NGO: Non-governmental organization; FLOT: Fluorouracil, leucovorin, oxaliplatin and docetaxel; CRT: Chemoradiotherapy Source references: [12, 18, 21, 25, 27].

Implementation gap	Locally adapted strategy	Purpose
Linguistic diversity	Record residence and preferred language at registration; create interpreter/counsellor pools for common catchment languages.	Enhances symptoms reporting, ensures informed consent, improves understanding of treatment, facilitates toxicity reporting, and strengthens follow-up processes.
Difficulty navigating tertiary centres	Appoint patient navigators/cancer-care coordinators for endoscopy, imaging, oncology OPD, PM-JAY desk, RT simulation, chemotherapy, and surgical referral.	Navigation programs can address logistical, financial, informational, and system barriers.
Repeated travel from remote/hilly areas	Use cluster scheduling: same-week biopsy, CT/PET-CT planning, nutrition review, MDT decision, and treatment plan.	Reduces repeated travel, lodging costs, missed visits, and delays.
Diagnostic delay	Create a Dysphagia-to-Treatment Fast Track for progressive dysphagia or suspected esophageal malignancy.	Prioritizes endoscopy, biopsy reporting, CT staging, nutrition assessment, and oncology review.
Accommodation and food burden	Establish low-cost accommodation and subsidized meal programs near cancer centers, in collaboration with hospitals, government agencies, and NGOs.	Addresses hidden indirect costs during prolonged CRT or chemotherapy.
Poor nutrition at presentation	Implement nutrition-first assessment: weight loss, dysphagia grade, hydration, dietician review, and feeding support when required.	Improves readiness for CRT, chemotherapy, surgery, and recovery.
Loss between departments	Conduct weekly esophageal MDT with radiation oncology, medical oncology, surgery, radiology, pathology, nutrition, palliative care, and the scheme/social-work desk.	Reduces fragmented decisions and loss during modality transitions.
PM-JAY/package approval delay	Create a dedicated cancer-package helpdesk for pre-authorization, documentation, treatment-plan approval, and appeal support.	Cancer packages require approval because treatment is multimodal.
Guideline expansion beyond package coverage	Update public packages to include biomarker testing, immunotherapy where indicated, FLOT-related support, feeding procedures, advanced staging, and toxicity management.	Aligns package coverage with contemporary evidence.
Follow-up and post-neoadjuvant tracking	Assign reassessment dates before neoadjuvant therapy ends; use phone/tele-follow-up and district-hospital linkage.	Prevents loss of surgical window, adjuvant therapy delay, and follow-up default.

Evidence from Meghalaya indicates that geographic remoteness, travel burden, financial hardship, caregiver constraints, and limited healthcare access can adversely affect engagement with cancer services [14,24]. These observations support implementation risk profiling at registration, including assessment of residence, travel time, preferred language, caregiver availability, insurance status, nutritional risk, affordability of stay, and ability to attend daily radiotherapy. Such an approach may facilitate early identification of patients at risk of treatment delays, interruptions, or loss to follow-up.

For tertiary care centres and regional cancer centres in North East India, a dysphagia-to-treatment fast track is a feasible local intervention. Once esophageal cancer is suspected, endoscopy, biopsy reporting, CT staging, nutritional assessment, and oncology review should be prioritized because every delay may worsen dysphagia, weight loss, performance status, and treatment fitness.

Community-engagement strategies should be culturally sensitive and leverage existing community structures. Given their influence and trust within many communities, traditional healers and indigenous practitioners could be engaged in cancer awareness initiatives and referral pathways [24, 28]. Training such stakeholders to recognize alarm symptoms, including progressive dysphagia, weight loss, and persistent upper gastrointestinal symptoms, may facilitate earlier presentation and referral to formal healthcare services. Patient navigation, social support screening, community engagement, and culturally adapted communication tools may improve timely and equitable cancer care delivery in North East India [22, 24, 25, 29].

Future directions

Future work should focus on measuring and reducing pathway attrition in esophageal cancer care. Tertiary and regional cancer centres should develop prospective implementation registries that capture stage, treatment, outcome, waiting time for endoscopy, biopsy, staging, MDT review, treatment initiation, radiotherapy completion, surgery, adjuvant therapy, follow-up, travel distance, language preference, nutritional status, and scheme coverage.

A locally adapted dysphagia-to-treatment fast track should be evaluated using practical endpoints such as diagnosis-to-treatment interval, treatment completion, radiotherapy interruption, post-neoadjuvant surgery rate, follow-up retention, and patient-reported financial burden [14, 27]. Future care models should formally integrate patient navigation, language-sensitive counselling, nutrition-readiness assessment, tele-follow-up, district-to-tertiary linkage, and social-support screening. Social support screening should extend beyond clinical staging to evaluate caregiver availability, travel distance, accommodation needs, language preferences, financial vulnerability, employment-related constraints, and access to government insurance schemes. Experiences during the COVID-19 pandemic highlighted the need for resilient oncology care pathways, as interruptions and treatment delays can adversely affect long-term outcomes among vulnerable patient populations [30]. 

At the policy level, government-supported oncology packages should be periodically aligned with current evidence, including biomarker testing, FLOT-based chemotherapy support, immunotherapy where indicated, feeding procedures, advanced staging, and toxicity management. In addition to improving healthcare delivery, future research should focus on generating region-specific molecular and biomarker data. Such efforts may facilitate risk stratification, biomarker discovery, and the development of more personalized treatment approaches in high-incidence populations [17, 31].

## Conclusions

Guideline-based management of esophageal carcinoma now requires coordinated staging, nutrition assessment, MDT decision-making, multimodality treatment, palliation, and follow-up. However, in North East India, the delivery of this pathway is often limited by delayed presentation, malnutrition, difficult travel, language barriers, financial burden, fragmented referral systems, package limitations, and loss to follow-up.

Improving care requires locally adapted implementation strategies, including fast-track dysphagia referral, nutrition-first assessment, structured MDT review, patient navigation, language-sensitive counselling, affordable stay and food support, district-to-tertiary linkage, and expansion of government-supported treatment packages. The key challenge is not only what guidelines recommend but also whether evidence-based care can reach patients in time, in full, and affordably.
